# Lithium Receipt and Risk of Dementia in Older Veterans With Bipolar Disorder in the VA Health System

**DOI:** 10.1093/geroni/igab046.2078

**Published:** 2021-12-17

**Authors:** Benjamin Szymanski, Eileen Ahearn, Eric Smith, Jenefer Jedele, John McCarthy, Ira Katz

**Affiliations:** 1 Department of Veterans Affairs, Ann Arbor, Michigan, United States; 2 William S. Middleton Memorial Veterans Hospital, Madison, Wisconsin, United States; 3 Edith Nourse Rogers Memorial Veterans Hospital, Bedford, Massachusetts, United States; 4 Veterans Health Administration, Ann Arbor, Michigan, United States; 5 Department of Veterans Affairs, Philadelphia, Pennsylvania, United States

## Abstract

Older adults with bipolar disorder are at increased risk of developing dementia. The literature suggests lithium treatment may reduce the incidence of dementia. This study sought to inform clinical practice in the Veterans Affairs (VA) health system by estimating the effect of past year lithium receipt on dementia incidence among Veterans with bipolar disorder. Divalproex receipt was used as a comparison. Using VA medical records, 121,094 Veterans aged 50 and older with a diagnosis of bipolar disorder but no dementia diagnosis were identified in fiscal years 2005-2019. Follow-up continued until dementia diagnosis, 36 months from the index date, death, or the end of fiscal year 2020, whichever came first. 4347 (3.6%) were diagnosed with dementia during follow-up. Time-varying indicators of receipt of lithium and divalproex in the prior 365 days were calculated for each day, categorized as 301-365, 61-300, 1-60, or 0 days of receipt. Unadjusted Cox proportional hazards regression analyses indicated reduced dementia incidence with 301-365 (HR=0.86, 95% Confidence Interval [95%CI] 0.75-0.99) and 61-300 (HR=0.75, 95%CI 0.65-0.87) days of lithium receipt, compared to 0 days. For divalproex, 301-365 (HR=1.34, 95%CI 1.23-1.47) and 61-300 (HR=1.13, 95%CI 1.03-1.23) days of receipt were each associated with increased dementia incidence. Lithium effects were not statistically significant after adjusting for age, sex, race, ethnicity, medical comorbidities, and antidepressant, antipsychotic, and anxiolytic medication receipt. Divalproex effects remained statistically significant. Past year divalproex, but not lithium, receipt was significantly associated with dementia incidence among VA patients with bipolar disorder when adjusting for demographics and medical comorbidities.

